# The Gain-Time Constant Product Quantifies Total Vestibular Output in Bilateral Vestibular Loss

**DOI:** 10.3389/fneur.2018.00396

**Published:** 2018-06-11

**Authors:** Timothy C. Hain, Marcello Cherchi, Nicolas Perez-Fernandez

**Affiliations:** ^1^Chicago Dizziness and Hearing, Chicago, IL, United States; ^2^Department of Neurology, Northwestern University, Chicago, IL, United States; ^3^Department of Otorhinolaryngology, Clinica Universidad de Navarra, Madrid, Spain

**Keywords:** bilateral vestibular loss, vestibular testing, rotatory chair, caloric testing, VHIT testing

## Abstract

Patients with inner ear damage associated with bilateral vestibular impairment often ask “how much damage do I have.” Although there are presently three clinical methods of measuring semicircular canal vestibular function; electronystagmography (ENG or VENG), rotatory chair and video head-impulse (VHIT) testing; none of these methods provides a method of measuring total vestibular output. Theory suggests that the slow cumulative eye position can be derived from the rotatory chair test by multiplying the high frequency gain by the time constant, or the “Gain*Tc* product.” In this retrospective study, we compared the Gain*Tc* in three groups, 30 normal subjects, 25 patients with surgically induced unilateral vestibular loss, and 24 patients with absent or nearly absent vestibular responses due to gentamicin exposure. We found that the Gain*Tc* product correlated better with remaining vestibular function than either the gain or the time constant alone. The fraction of remaining vestibular function was predicted by the equation *R* = (Gain*Tc*/11.3) – 0.6. We suggest that the Gain*Tc* product answers the question “how much damage do I have,” and is a better measure than other clinical tests of vestibular function.

## Introduction

Patients with inner ear damage associated with bilateral vestibular impairment often ask “How much damage do I have.” They ask “Are my inner ears getting better or worse?” These questions can be difficult to answer because of our limited repertoire of vestibular tests. Although there are presently three clinical methods of measuring semicircular canal vestibular function; videonystagmography (VNG), rotatory chair and video head-impulse (VHIT) testing; none of these methods provides a method of measuring total vestibular output.

By total vestibular output we mean: the total ocular response for a given change in head velocity. This is not the same as the peak eye velocity, as the peak is a response at a particular time, that does not account for responses prior to and following the peak. The total response requires adding up all of the eye movement output over time. We will develop below the argument that a reasonable quantity to describe the total vestibular response is cumulative eye position.

One might argue that the caloric portion of VNG testing provides a “total response” parameter. However, this is a peak velocity measurement and thus is not a total response, which would require considering all eye movement elicited by the caloric stimulus. Furthermore, the caloric input corresponds to very low frequencies of vestibular stimulation, analogous to 0.003 Hz (1). Thus, the VNG doesn't cover the entire frequency range of the vestibular response.

The VHIT device has no total vestibular response parameter. This is again because it measures velocity rather than position. Furthermore, the VHIT is limited to high frequencies of vestibular stimulation, predominantly at 2.5 Hz (2). According to Patel et al., the output of the VHIT, high frequency gain, is not correlated with chronic symptoms of dizziness (3).

The rotatory chair test assesses a broad range of input frequencies, typically 0.01–0.64 Hz, and thus provides more information than either the caloric or the VHIT. However, the rotatory chair test does not provide a “total vestibular response” parameter. We will discuss how this can be computed.

The rotatory chair quantifies the velocity gain and phase of the vestibular-ocular reflex (VOR) in the horizontal plane. These values are obtained typically for at least four frequencies ranging from low (0.01 Hz) to high (0.64 Hz). The gain and phase values are plotted against the range of normal, and inferences are made concerning vestibular function from their pattern. As frequency ranges from high to low, individuals with peripheral vestibular disorders exhibit both reduced gain and increased phase, but there is considerable variability (4).

While recognition of specific patterns of gain and phase of rotatory chair plots is useful, it is often imprecise and it can also be challenging to explain to patients that their problem is “phase lead at low frequencies.” Fortunately, the gain and phase plots such as are used in rotatory chair testing have a descriptive mathematics that can be used to simplify this situation. The dependence of output on input can be expressed as a “transfer function.” For a linear system, the output for any stimulus can be predicted from the transfer function. As the vestibulo-ocular reflex (VOR) is largely linear for low velocities and accelerations (5), its transfer function can be represented by a linear mathematical construct, which for the VOR is a “single pole.” The single pole has two parameters—gain (K) and time constant (*Tc*) (6). Although having only two values (gain and time constant) is simpler to interpret than the eight values contained within the gain/phase frequency plots, the gain, and time constant values do not answer the question often posed by patients with bilateral vestibular impairment: “How much of the vestibular system remains?”

A possible solution lies in the “Slow cumulative eye position,” or SCEP. Equations (1) and (2) express the mathematics underlying the SCEP. This transfer function for the vestibulo-ocular reflex can be computed from the step response—an exponentially declining eye velocity that is produced by a sudden change, or “step” in head velocity. The SCEP is the integral of the VOR step response and represents the total angular displacement of the eye for a step of head velocity. Thus, the SCEP reflects total vestibular output, in units of ocular angular displacement, for a step change of head velocity. When the step response equation is normalized to a 1°/s step (Equation 1) and then is integrated over time (Equation 2),the total eye displacement is simply the product of K ^*^ *Tc*—the product of the gain and the time constant. We subsequently call this the “Gain*Tc*.”

Equation 1: Eye velocity in response to a unit 1°/s step of head velocity.

Ė = eye velocity, *K* = High frequency gain, *t* = time, and *Tc* = Time constant:

(1) $$\begin{array}{l} Ė=K\times e^{-t/Tc} \end{array}$$

Equation 2: Slow cumulative eye position, or SCEP

(2) $$\begin{array}{l} E=\int Ė=\int K\times e^{-t/Tc}=K\times Tc \end{array}$$

The SCEP is computationally straightforward, being simply the product of the gain and time constant, and from Equations (1) and (2), one can see that this single number represents the total output, in terms of cumulative eye position, for a unit 1°/s step of head velocity.

For a group of subjects in whom the amount of remaining vestibular function was known we asked the question: How well does the Gain*Tc* correlate with remaining vestibular function?

## Methods

This was a retrospective study in which we computed the Gain*Tc* in three groups of subjects. The first group had good evidence for normal vestibular function. The second group had surgical unilateral hypofunction. The third group had near complete bilateral vestibular loss due to exposure to the ototoxic antibiotic, gentamicin. They are described in more detail below.

This study was reviewed and approved by the Northwestern University Institutional Research Board. A waiver of consent was granted for this retrospective review of data.

Most subjects were derived from the clinical practice of the first two authors (TCH, MC). These subjects underwent rotatory chair testing on a Micromedical Technology Rotatory chair system (Model 2000, GN-Otometrics, Chatham, Illinois), with a peak acceleration of 200°/s^2^. Fifteen of the 25 surgical subjects were contributed by the third author (NPF). These subjects were tested on a similar device, the CHARTR® RVT system, ICS Medical Corporation, Schaumburg, IL). All subjects were tested using standard protocols that included sinusoidal stimulation up to 0.64 Hz, and step responses. The parameters used to compute the Gain*Tc* were produced by the commercial software for these two devices.

The VOR gain (*K* in Equations 1 and 2) was computed from the ratio of peak eye velocity/peak head velocity) for sinusoidal rotatory chair testing at 0.64 Hz, which is the highest frequency used for routine rotatory chair testing with these devices. The time constant, *Tc* was taken from the average computed time constant for per and post-rotatory measurements of two 100°/s step responses. The frequency of 0.64 Hz was chosen because it is the highest frequency available for these tests, and because while the VOR gain depends on frequency, it asymptotes to a constant level at higher frequencies (7).

## Results

Group 1, “normal,” included 30 subjects. Six of these were normal volunteers with no complaints of dizziness or hearing disturbance. The remaining 24 complained of dizziness but had entirely normal otoneurological examinations including bedside video Frenzel testing. The bedside exam included negative testing for spontaneous nystagmus, vibration induced nystagmus, and head-shaking nystagmus, and a negative eyes-closed tandem Romberg. This testing is very sensitive to unilateral and bilateral vestibular loss. In 21 of the “normal” subjects, the cause of their dizziness was attributed to migraine as they also had headaches as a prominent feature. In the remaining three, final diagnoses were epilepsy, anxiety, and syncope. Fifteen of these subjects also had caloric testing done. These subjects had normal caloric responses using conventional criteria (1). For patients where it was available, the average total caloric response was 76.11°/s. We assumed that these subjects had 100% of their vestibular system functioning and set the *R*-value to 1.

Group 2, “unilateral,” included 25 subjects. Of these, 21 had undergone surgery to ablate vestibular function on one side. Six of these had vestibular nerve sections, and 15 were post labyrinthectomy. The remaining four had surgical removal of large acoustic neuromas followed by a caloric test that documented no remaining vestibular function. Twenty-four of these subjects had caloric testing done, and all but three had no caloric response on the operated side. In these three, the caloric paresis was very high (79, 91, 93%). In these cases, we accounted for the remaining residual function. Specifically, we assumed the remaining ear had an “*R*” of 0.5, and we solved the paresis equation of Jongkees (8) for the other ear. These adjustments were small and resulted in a mean value for “*R*” of 0.51, only slightly greater than the expected 0.5 for surgical lesions.

Group 3, “bilateral,” included 24 patients with bilateral vestibular loss. These individuals had developed permanent oscillopsia and ataxia after exposure to gentamicin, a well-known ototoxin. Twenty-two of twenty-four of these had caloric responses available, and the average total caloric response was 10.05°/s. Rather than assume that they had no vestibular function at all, in the 22 where caloric results were available, we estimated their remaining vestibular function by dividing the sum of all four open water caloric irrigations by 100. The figure of 100 was chosen as it is the average sum of all caloric irrigations of normal persons to open water caloric testing (9). While this is called the “total caloric response,” here the term “total” refers to an aggregate descriptor of the conventional caloric test, rather than the entire output of the caloric test (which would require an integral). As previously observed by Hess et al. (10), it is likely that the caloric underestimates the true remaining vestibular function, in as much as caloric testing is a test of low frequencies, and cannot assess the higher frequency VOR. Nevertheless, considering the lack of better data this is the most reasonable adjustment.

Table 1 shows that the average Gain*Tc*-value varied greatly between normal (11.25), unilateral (3.75), and bilateral (0.95) groups. As the values of the Gain*Tc* parameter were not normally distributed for each group we used the Kruskal-Wallis test to compare group differences (11). There was a statistically significant difference in Gain*Tc* parameter for the three groups [*H*_(2)_ = 63.657, *p* < 0.001], with a mean rank of 64.15 for the normal group, 35.42 for the unilateral group, and 14.58 for the bilateral group. Follow up pairwise comparisons revealed that the Normal group was significantly higher than both the unilateral (*H* = 28.730, *p* < 0.001) and bilateral-gent (*H* = 49.567, *p* < 0.001) groups, and the Gain*Tc* parameter was higher for the unilateral group than for the bilateral group (*H* = 20.837, *p* = 0.004).

**Table 1:** Summarizes the characteristics of the three groups of subjects as well as provides summary values for vestibular parameters from rotatory chair testing.

	***n***	***R***	**Gain**	***Tc***	**Gain*Tc***	**Age**
Normal	30	1.00	0.75 ± 0.11	15.28 ± 5.34	11.25 ± 3.13	36.07 ± 12.9
Unilateral	25	0.50	0.51 ± 0.15	7.38 ± 2.76	3.75 ± 1.51	52.16 ± 10.0
Bilateral	24	0.09	0.35 ± 0.21	3.22 ± 1.28	0.95 ± 1.18	62.08 ± 11.11

*n = number of subjects. Remaining function (R) = fraction of total vestibular function remaining (see text). Gain is the VOR gain for 0.64 Hz. Tc is the average time constant for step responses. GainTc = product of VOR Gain and Tc. Standard deviations are provided next to the mean values*.

**Figure 1:**
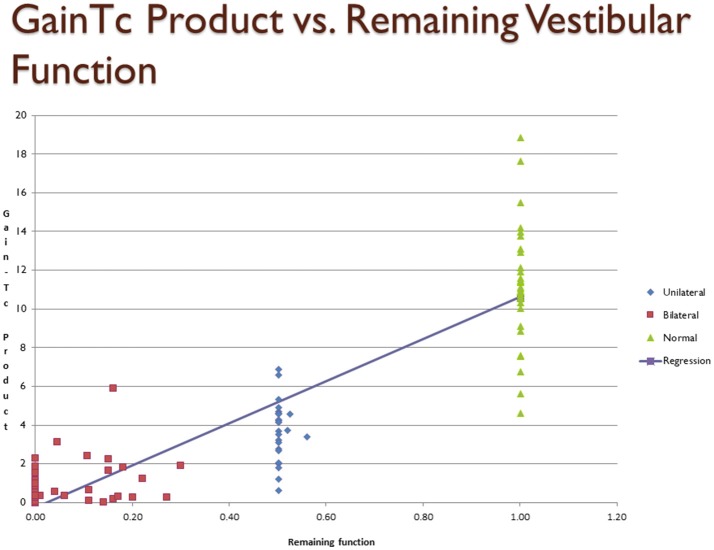
Scatter plot showing inferred remaining vestibular function, on the X axis, against the Gain*Tc* product on the Y axis. Group 1 = normal subjects. Group 2 = patients with surgical unilateral vestibular loss. Group 3 = patients with bilateral vestibular weakness on caloric testing caused by gentamicin ototoxicity. The regression shows the line fit between remaining vestibular function and the Gain*Tc* product.

Figure 1 is a scatter plot showing remaining vestibular function, on the X axis, plotted against the Gain*Tc* on the Y axis. The linear regression line shown in Figure 1 is described in Table 2.

**Table 2:** Regressions of Gain*Tc*, Gain, and *Tc* on remaining vestibular function, *R*.

**Parameter**	***r^2^***	**Slope**	**Intercept**
Gain*Tc*	0.76	11.3	−0.60
Gain	0.55	1.23	0.11
*Tc*	0.60	0.045	0.15

Each of these regressions can be used to estimate the remaining function based on the GainTc, Gain, or Tc. For example, using the GainTc regression, GainTc = (11.3 * R) – 0.6. More usefully, when rearranged in terms of remaining function, the equation becomes R = (GainTc/11.3) – 0.6.

## Discussion

Our results suggest that the mean Gain*Tc*—the product of the VOR gain and time constant, differs significantly between the normal, unilateral loss and bilateral loss groups, and furthermore the Gain*Tc* correlates with remaining vestibular function. In particular, a reasonable estimate for the remaining fraction of vestibular function that an individual has, which we call “*R*,” neglecting the small Y intercept, is the product of their VOR Gain and step-response time constant, computed as we described, divided by 11.3.

We have also shown that the correlation of Gain*Tc* with *R* is higher than either the VOR gain or the VOR time constant, *Tc*. Based on this analysis, we suggest that the Gain*Tc* product is a better measure of total vestibular function than either Gain or *Tc*. Because, as we have shown, the Gain*Tc* is proportional to the total vestibular output, the Gain*Tc* is also the measure most suitable to quantify bilateral vestibular damage such as resulting from ototoxicity. As mentioned in the introduction, the other tests of semicircular canal function contain less information than the rotatory chair because they assess low frequencies (i.e., caloric tests) alone, or primarily very high frequencies (i.e., VHIT test).

Although the Gain*Tc* product is not a complex construct, we were able to find only a single other mention of this parameter in the vestibular testing literature. Honrubia et al. (12), when describing the results of rotatory chair testing in subjects with bilateral vestibular weakness, reported the “coefficient of sensitivity of the pendulum equation,” that was computed identically. They stated that the value for this parameter was 5.87 in normal subjects, while the mean value for the nine patients with bilateral vestibular weakness that they reported was 1.60. This result is smaller than ours, possibly because of their use of smaller values for the VOR time constant based on higher velocity rotational tests than those used in the present work. Honrubia et al. did not develop this concept further.

Other estimates of the Gain and *Tc* and their product can be obtained from larger studies of rotatory chair testing in patients.

Table 3 summarizes literature data, selecting out those having larger numbers of subjects, and providing gain and *Tc*-values from rotatory chair testing in normal subjects and patients with well documented unilateral vestibular loss. For normal subjects, the study of Wade et al. (13), included almost an order of magnitude more subjects than other similar studies, and computing the Gain*Tc* from their data resulted in a similar estimate for the “normal” Gain*Tc* (10.4) as ours (11.3). Average gains and time constants from smaller studies (4, 14–17), yielded values for the Gain*Tc* product ranging from 7.12 to 11.26.

**Table 3:** Literature values for gain and Tc.

***n***	**Gain**	***Tc***	**Gain*Tc***	**References**
**NORMAL SUBJECTS**				
55	0.53	13.44	7.12	(14)
743	0.65	16.00	10.4	(13)
100	0.66	16.6	11.0	(15)
**UNILATERAL VESTIBULAR LOSS**				
7	0.49	6.2	3.04	(18)
43	0.38	9	3.38	(13)
11	0.49	6.2	3.04	(19)

With respect to studies reporting values for patients with well documented unilateral loss, all of the studies in Table 3 result in a similar estimate for the Gain*Tc* product, between 3.03 and 3.375. This is similar to our finding of a value of 3.75 in 25 subjects.

For patients with bilateral vestibular loss, if their vestibular loss is complete, the gain would be 0 and thus the Gain*Tc* product should be 0. We know of no datasets comparable to ours where caloric responses were used to estimate total response in patients with gentamicin induced ototoxicity. There is, however, documentation of the failure of individual gain and *Tc*-values to correlate with reduction of caloric responses. Hess et al. reported a wide range of gains (0–0.8) and time constants (1–9) in 17 patients with partial bilateral vestibular loss (10).

As the ages of our unilateral and bilateral subjects were much lower than those of our normal subjects, and one might hypothesize that the effects seen were at least partially due to decline in vestibular output with age. We computed the Gain*Tc* product from several other studies of the VOR as a function of age (20, 21) in an attempt to estimate the magnitude of the effect of age on the Gain*Tc* product.

Baloh et al. reported the gains and time constants of the VOR in a study of 75 “elderly” normal people, averaging 79.6 years old, who were compared to 25 normal younger people, averaging 26.2 years old (20). Their data results in a Gain*Tc* product of 9.5 for the younger subjects, and 6.8 for the older subjects, from which it can be calculated that the slope of the Gain*Tc*/year is−0.051/year. Similarly, Paige (21) reported gain and phase data in 30 “young” (18–44) and 23 “Middle-Aged” (45–69) subjects. The Gain*Tc* products for the 0.025 Hz−50°/s stimulus, similar to our methodology, were computed to be 13.23 and 10.07 for young and middle-aged, respectively, resulting in a slope of −0.12°/year. This analysis suggests that the Gain*Tc* product declines slowly with age. From these two values, for the ages of our subjects, the amount of decrement of Gain*Tc* that would be predicted from the age differences between our bilateral (average age of 62) and normal subjects (36) is between 1.3 and 3.1°. This amount of decline in the Gain*Tc* is much smaller than the 7.5° or more deg. decline in Gain*Tc* found in the unilateral and severe bilateral vestibular groups.

As the Gain*Tc* product has the highest correlation (*r*^2^), with remaining function, this implies that it better reflects remaining vestibular function than high-frequency VOR gain or the time constant. We suggest that the Gain*Tc* product performs better because of the following observations: As illustrated by Table 3, compared to normal subjects, vestibular gain is little changed by unilateral vestibular weakness or loss. However, the time constant is greatly reduced. In patients with near complete vestibular loss, the VOR time constant cannot decrease below that of the mechanics of the inner ear (about 6 s) (6), but the gain continues to decrease until it reaches 0. Thus, the Gain*Tc* product, which is sensitive to unilateral loss from decline in the time constant, and is sensitive to bilateral loss from decline in the gain, correlates better with remaining vestibular function than either the gain or the time constant, considered separately. Furthermore, as the Gain*Tc* product is a continuous variable, it could be reasonably used to follow progress over time and answer questions such as: Am I getting better or worse?

## Limitations

Our “normal” group was largely composed of individuals with complaints of dizziness, but with no peripheral vestibular lesion. It is possible that these persons had undiscovered peripheral vestibular lesions, or the process that caused their symptoms affects the Gain*Tc* Product. While possible, this is unlikely, as in Table 3 we point out that several large studies of normal subjects containing data from which the Gain*Tc* product can be computed, produce similar values to ours.

Second, participants in this study were tested in two different commercially available rotatory chairs. It is possible that there are systemic differences in the Gain*Tc* product, depending on differences in device characteristics. Again, while possible, this is unlikely as in Table 3 we point out that studies of subjects in many other settings, including subjects with unilateral vestibular loss, resulted in similar values for the Gain*Tc* product.

Thirdly, the Gain*Tc* product is a measure of horizontal canal function alone. It does not quantify vestibular function of the vertical semicircular canals or the otolith organs. Other vestibular tests such as the VHIT or caloric test are better able to determine the side of a vestibular lesion. This is an important consideration that shows that the rotatory chair test quantifies only a subset of vestibular function. Nevertheless, the Gain*Tc* parameter, appears well suited to for quantification of bilateral vestibular weakness that affects the entire vestibular apparatus.

## Conclusion

The Gain*Tc* product is a method of inferring remaining vestibular function of the horizontal semicircular canals. It provides an answer to the question “how much vestibular function do I have.” It suffers from the variability intrinsic to other vestibular measures, but has the advantage of simplicity, as it provides a “single number” to quantify vestibular output. As it is a continuous variable, it may be suitable to monitoring progressive vestibulopathies such as those commonly encountered in ototoxicity.

## Author contributions

TH accumulated the data, wrote the manuscript. MC reviewed the manuscript. NP-F contributed cases from his practice and provided critical review of the manuscript.

### Conflict of interest statement

The authors declare that the research was conducted in the absence of any commercial or financial relationships that could be construed as a potential conflict of interest.
